# Retrospective analysis of secreted PrpL protease activity in clinical isolates of *Pseudomonas aeruginosa* and its association with corneal tissue damage

**DOI:** 10.3389/fmicb.2026.1824817

**Published:** 2026-05-08

**Authors:** Douglas S. Parker, Onyedikachi C. Azuama, Kashaf Zafar, Jon D. Goguen, John M. Leong, Nikhat Parveen

**Affiliations:** 1 Department of Microbiology and Physiological Systems, University of Massachusetts Medical School, Worcester, MA, United States; 2 Department of Microbiology, Biochemistry and Molecular Genetics, Rutgers-New Jersey Medical School, Newark, NJ, United States; 3 Department of Molecular Biology and Microbiology, Tufts University School of Medicine, Boston, MA, United States

**Keywords:** corneal infections, *Pseudomonas aeruginosa*, pyocyanin, serine protease PrpL, tissue damage, type II secretion

## Abstract

**Background:**

*Pseudomonas aeruginosa* is a ubiquitous organism that adapts well in different environments. It is an opportunistic bacterial pathogen that produces a wide range of virulence factors, colonizes lungs to cause pneumonia, causes non-healing wounds especially in burn victims, and is a major culprit in destructive keratitis. It can reach the cornea through reusable, extended-use contact lenses and by contaminated eyedrops and artificial tears. Secreted proteases of *P. aeruginosa* together with pyocyanin metabolite, which inhibits Serine Protease Inhibitors (Serpins) activity contribute to severe tissue damage during infection.

**Methods:**

*Pseudomonas aeruginosa* strains isolated from different clinical sites were obtained from different researchers and clinicians for this study. We examined *P. aeruginosa* strains and secretion defective and prpL knockout mutants in PA64481 strain for lysyl endopeptidase (PrpL, a serine protease that cleaves after a lysine residue) activity using serine protease specific D-Val-Leu-Lys -p-nitroanalide substrate. We also determined pyocyanin production in these strains.

**Results:**

Examination of secreted milieu from *P. aeruginosa* showed that 41 corneal isolates had detectable lysyl endopeptidase activity associated with PrpL at levels significantly higher than by 27 non-corneal isolates. We found that PrpL is secreted by the *xcp-*based type II secretion system. Bacterial culture supernatants displaying higher PrpL activity and not low secreted PrpL levels disrupted corneal epithelial cell monolayers *in vitro,* which is consistent with a role of this protease in destructive keratitis. Many examined strains also produced high levels of pyocyanin.

**Conclusion:**

This retrospective examination of clinical *P. aeruginosa* suggests that high levels of PrpL and pyocyanin-producing isolates are more prevalent among corneal isolates and could enhance tissue damage during infection. Supporting this premise, corneal epithelial cell monolayers disrupted by high PrpL-producing strains but remained intact after treatment with *P. aeruginosa* mutants culture supernatants that lack or have reduced secreted PrpL.

## Introduction

*Pseudomonas aeruginosa* is a ubiquitously existing Gram-negative bacterium which is also an important human pathogen. *P. aeruginosa* can produce lethal infections in mice, flies, nematodes, and plants, making it the pathogen *par excellence* (Hendrickson et al., 2001; Letizia et al., 2025). In humans, it is an opportunist that requires tissue damage and/or weakened host defenses to produce significant disease. Consistent with its adaptation to parasitism, *P. aeruginosa* utilizes type III secretion system to directly inject protein effectors into the cytoplasm of eukaryotic cells and employs other secretion systems to export several virulence factors into the environment. *P. aeruginosa* infection often causes non-healing wounds especially in burn victims and inflicts acute to chronic lung disease in the intensive care units and among cystic fibrosis patients. It is a primary contributor in corneal infections and ulcerative keratitis particularly among extended-wear contact lens users (Lim et al., 2016; Mukhtar et al., 2022; Musa et al., 2010). Furthermore, immunocompromised and post-cataract surgery individuals are predisposed to *P. aeruginosa* infection (Parchand et al., 2020). Emergence of multidrug-resistant (MDR) and extensively drug-resistant (XDR) *P. aeruginosa* strains has greatly complicated treatment of these infections. A recent outbreak of MDR/XDR *P. aeruginosa* corneal infections due to contaminated artificial tear eye drops indicates that this infection can result in blindness and even death due to sepsis (Morelli et al., 2023; Rezaei et al., 2023; Sundermann et al., 2024), thus needing better understanding of the critical virulence factors of *P. aeruginosa*.

*Pseudomonas aeruginosa* secretes several degradative proteases into the extracellular environment (Fortuna et al., 2024; Okamoto et al., 1997). Among these, alkaline protease, elastase A, and elastase B are firmly implicated in *P. aeruginosa* pathogenesis in cystic fibrosis patients (Toder et al., 1994) but not in keratitis (Hobden, 2002). Proteases degrade components of extracellular matrix (ECM), causing collagen fibrils to disperse, weakening the corneal stroma and leading to subsequent corneal perforation under anterior chamber pressure (O'Callaghan et al., 2019). Mammalian protease inhibitors regulate the activity of host, and several *P. aeruginosa* proteases, potentially minimizing tissue injury they cause. The fourth, a ~ 27 kD serine protease labeled as Protease IV (Engel et al., 1998a; Engel et al., 1998b) was previously purified from PA103-29 strain and implicated in ulcerative keratitis in an experimental animal model (Caballero et al., 2001; Engel et al., 1998a; Engel et al., 1998b; Engel et al., 1997; Malloy et al., 2005; O'Callaghan et al., 2019; Traidej et al., 2003). PrpL significantly enhances corneal damage by degrading complement components, immunoglobulins, surfactant proteins, and extracellular matrix substrates (Caballero et al., 2001; Engel et al., 1998a; Engel et al., 1998b). Also, PrpL promotes excessive neutrophilic inflammation, thereby aiding tissue damage and corneal opacity. The virulence of PrpL is not restricted to mammalian cornea; it is equally involved in the damage of the antibacterial peptide inducer IL-22 (Cytokine-Interleukin-22), and respiratory surfactant proteins. This serine protease/PrpL is highly specific for peptide bonds at the carbonyl group of lysine residue (lysyl-endopeptidase) in protein, ester, or amide substrates. Therefore, D-Val-Leu-Lys-p-nitroanalide or S2251 chromogenic substrate is ideal for PrpL activity determination.

Complex mechanisms regulate the expression of different virulence factors of *P. aeruginosa*. PvdS (PA2426 in PA01 strain) is an iron-starvation regulator that drives the expression of genes involved in the synthesis of the pyoverdine siderophore (Imperi et al., 2010) and extracellular protease PrpL (PvdS-regulated endopeptidase, lysyl class)/Protease IV and the secreted exotoxin A (Wilderman et al., 2001). Activities of serine proteases are regulated in hosts largely through production of a group of protease inhibitors, such as the serpins (*Ser*ine *p*rotease *in*hibitors) class (O'Connor et al., 1993; Potempa et al., 1994). Among the most prominent anti-proteases is α1-protease inhibitor (α1-PI), which forms an enzymatically inactive complex with various serine proteases and may also control host proteases activities (Potempa et al., 1994; Travis and Salvesen, 1983). Anti-proteases could similarly limit the damage caused by bacterial serine proteases, such as PrpL. Interestingly, proteases also play critical roles in *P. aeruginosa* competitiveness and fitness in hosts by cleaving host serpins, enhancing shedding of host proteins to increase invasiveness and colonization in cornea and airway in cystic fibrosis patients (Aristoteli and Willcox, 2003; Rapala-Kozik et al., 1999).

*Pseudomonas aeruginosa* has adapted to the potentially antagonistic effects of host protease inhibitors like α1-PI on bacterial proteases by also secreting pyocyanin, a phenazine compound that counteracts host protease inhibitors. Oxidation of methionine at the active site of α1-PI results in inactivation of this inhibitor (Desrochers et al., 1992). Pyocyanin stimulates production of oxidants such as superoxide (O2^.-^), and hydrogen peroxide (H_2_O_2_) under aerobic conditions and in the presence of reducing compounds such as NADH, it could also control the intracellular redox homeostasis in bacteria (Britigan et al., 1992) and hosts. Pyocyanin-treated α1-PI failed to inactivate the serine proteases and the inhibitor was then degraded by either trypsin or porcine pancreatic elastase *in vitro* (Britigan et al., 1999). Thus, pyocyanin and PrpL could synergistically exacerbate tissue damage by *P. aeruginosa* especially during corneal and lung infection.

In this study, we show that consistent with a role in keratitis, PrpL activity in human corneal *P. aeruginosa* isolates was significantly (*p* = 0.035) higher than that in non-corneal clinical isolates and PrpL was associated with the destruction of cultured corneal epithelial cell monolayers not observed in *xcpA* and *prpL* mutants. Based upon literature and our results here, we anticipate that pyocyanin, an antagonist of serpins, would augment the activity of PrpL and exacerbate tissue destruction, highlighting the importance of proteolytic damage in the pathogenesis of *P. aeruginosa* in humans.

### Ethics statement

Patients personal information, clinical details and other information associated with *P. aeruginosa* strains were not provided except documentation of the original isolation site and strain numbers allocated. Therefore, informed consent from patients is not applicable for this study. However, corresponding author has approved exempt Institutional Protocol Board protocol to conduct studies with clinical bacterial isolates.

## Materials and methods

### Bacterial strains, corneal cell line, and growth conditions

Several *P. aeruginosa* strains including PA01 and PA103 were generously provided by Dara Frank. Other corneal and non-corneal strains, including PA64481, were gifts of Darlene Miller of Bascom Palmer Eye Institute and Brenda Torres of the University of Massachusetts-Memorial clinical laboratories. Independent isolates from different patients visiting the facilities over time were collected by clinicians and labeled strains provided to us for our studies.

*Pseudomonas aeruginosa* cultures were grown in iron sufficient Luria Bertani (LB) or in SM9 minimal medium containing 2 g Glucose, 3 g KH_2_PO_4_, 6 g Na_2_HPO_4_, 1 g NH_4_Cl, 5 g NaCl, 0.2 μM CaCl_2_, 1 μM MgSO_4_ and 1 mg Thiamine per liter, pH 6.9. Human corneal epithelial cell line developed by Araki-Sasaki by transformation with SV40 vector (HCE-T), was obtained from RIKEN, Japan. The HCE-T cell line was cultured in the medium containing DMEM: Ham12 in a 1:1 ratio, 5%FBS, 5 μg/mL insulin, 0.1 μg/mL cholera toxin, 10 ng/mL hEGF, and 0.5%DMSO at 37 °C in 5% CO_2_ incubator.

### Analysis of PrpL protease activity and determination of pyocyanin levels

We assayed PrpL protease (PA4175) activity using chromogenic plasmin substrate D-Val-Leu-Lys -p-nitroanalide or S2251 from Sigma Chemical Company (O'Callaghan et al., 2019) and kinetic studies carried out in microtiter plates at 37 °C. In a 100 μL reaction volume with 85 μL buffer, containing 50 mM Tris, pH 7.56, 0.1% Tween 80, 0.02% Sodium azide, 1% glycerol, 5 μL culture supernatant and 10 μL substrate (5 mM final) added per well. Serine proteases, including PrpL, specifically cleave the chromogenic peptide substrate S2251 (D-Val-Leu-Lys-p-nitroanilide), releasing p-nitroaniline producing yellow color. The formation of p-nitroaniline is measured as an increase in absorbance at 405 nm, providing a direct, quantitative measure of PrpL enzymatic activity (p-nitroaniline absorbs maximally at 405 nm) (Caballero et al., 2001b; Engel et al., 1998a). The reproducibility of the experiment was ensured by performing each assay in triplicate and repeating the experiment independently at least three times. Total pyocyanin levels in culture supernatants were extracted with chloroform, followed by acidification with 0.2 N HCl, and quantified calorimetrically as previously described (Essar et al., 1990; Hendrickson et al., 2001). Briefly, bacteria were grown at 37 °C after inoculation as a 100-fold dilution of log-phase cultures in LB broth with shaking for 20 h. After growth, 1 mL of culture was extracted with 2 mL of chloroform and centrifuged at 12,000 × g for 5 min. The blue chloroform layer was transferred to a new tube containing 1 mL of 0.2 N HCl, which extracted pyocyanin into the acidic phase. The concentration of pyocyanin was determined by measuring the optical density at 520 nm (OD₅₂₀).

### Purification of PrpL protease and raising antibodies

PrpL was purified as previously described (Engel et al., 1998a), with minor modifications. Briefly, culture supernatant from the high PrpL-producing strain PA64481 grown in minimal medium was subjected to ion-exchange chromatography on DEAE-Sepharose, followed by size-exclusion chromatography. This two-step purification yielded a single major peak of activity corresponding to an estimated molecular mass of approximately 27 kDa. No other fraction showed significant PrpL activity. We used the pulverized PrpL protein band from PA64481 supernatant resolved in SDS-PAGE to raise polyclonal antibodies in mice.

### SDS-PAGE, silver staining, and Western blot analysis

Culture supernatants from a specific number of *P. aeruginosa* strains in each experiment were precipitated with 1/10 volume of 100% TCA, pellet washed with acetone and boiled with protein loading dye after resuspension of pellet in PBS. SDS-PAGE was conducted using culture supernatants (or lysates) followed by silver staining using silver stain plus kit (Bio-Rad catalog number 161–0449) or Western blot analysis using anti-PrpL polyclonal antibodies raised in mice, as described above.

### Mutagenesis and cloning of mutated DNA fragment

Random transposon mutagenesis of PA64481 strain was achieved by conjugational transfer of non-replicative plasmid pRK2013 containing Tn5 with gentamycin (Tn5Gm) from *E. coli* strain 1849(pRK2013: Tn5Gm), which is unable to synthesize diaminopimelic acid (DAP^−^). We selected mutant on casein overlay on LBGent^75^ plates lacking DAP and selected transposon-insertion mutant colony that lacked a zone of casein digestion. The site of transposon insertion in the mutant was determined by cloning the fragment in pGATA (Amp^r^) vector followed by sequence analysis (Stapleton et al., 1984). Directed mutagenesis of the *prpL* gene was performed by allelic exchange. Briefly, the *prpL* coding region together with ~300 bp upstream and downstream flanking sequences (~1.8 kb) was amplified by PCR and cloned into TopoXL suicidal vector. A gentamicin-resistance cassette to disrupt the gene was inserted within the *prpL* coding sequence using Age1 and Sal1 that deleted ~1 kb central region. The recombinant construct was introduced into *P. aeruginosa* PA64481 by electroporation and mutants were selected on gentamicin-containing plates. Transformants were screened by colony PCR and gene disruption (Δ*prpL*) was confirmed by PCR using isolated genomic DNA followed by sequencing (Hmelo et al., 2015; Hoang et al., 1998; Schweizer, 1991).

### Effect of *Pseudomonas aeruginosa* culture supernatants on HCE-T cell line

The HCE-T monolayers on coverslips were incubated with culture supernatant diluted 1:1 in the cell culture medium up to 1 h at 15 min interval, fixed with 3% paraformaldehyde prepared in phosphate buffered saline, and permeabilized with 0.1% Triton-X100 for 5 min. After washing three times with PBS at 5-min intervals, the cells were stained to detect cytokeratins by incubating with 1:100 dilution of anti-keratin guinea pig antibodies (Sigma Chemical, Inc.) for 1 h. After washing, coverslips were incubated with anti-guinea pig FITC (Sigma F6261) secondary antibodies together with TRITC-phalloidin (0.5 μg/mL) for 1 h for visualization of cytokeratins and filamentous actin, respectively. After mounting coverslips, we observed the slides with Nikon inverted fluorescence microscope.

### Statistical analysis

Each experiment was conducted at least three times with two replicates for each HCE-T cells treatment and three replicates for PrpL activity determination. Reproducible results and pattern were obtained in different experiments. Results from one representative experiment are shown in each figure. Mann–Whitney two-tailed U test for asymmetrically distributed data was used to compare PrpL activity levels in corneal versus non-corneal clinical *P. aeruginosa* isolates. Overall, PrpL activity was significantly higher (*p* < 0.05) overall in corneal isolates by this analysis. Fluorescence intensity associated with HCE-T monolayers were quantified from seven independent microscopic fields of view after treatment of cells with culture supernatant of each *P. aeruginosa* strain using ImageJ and presented as mean fluorescence intensity. Error bars in the Supplementary Figure S2 indicate Mean ± standard error of mean (SEM). SEM values are also listed in legends of Figures 3, 5, 6 of the manuscript. Statistical analysis was performed using one-way ANOVA, which revealed that PA64481 exhibited a significantly lower mean fluorescence intensity compared to the other strains (****p < 0.0001), mainly because of efficient destruction of the cell monolayer by secreted milieu of this strain.

## Results

### Supernatants from *Pseudomonas aeruginosa* corneal isolates exhibit higher PrpL activity

**Figure 1:**
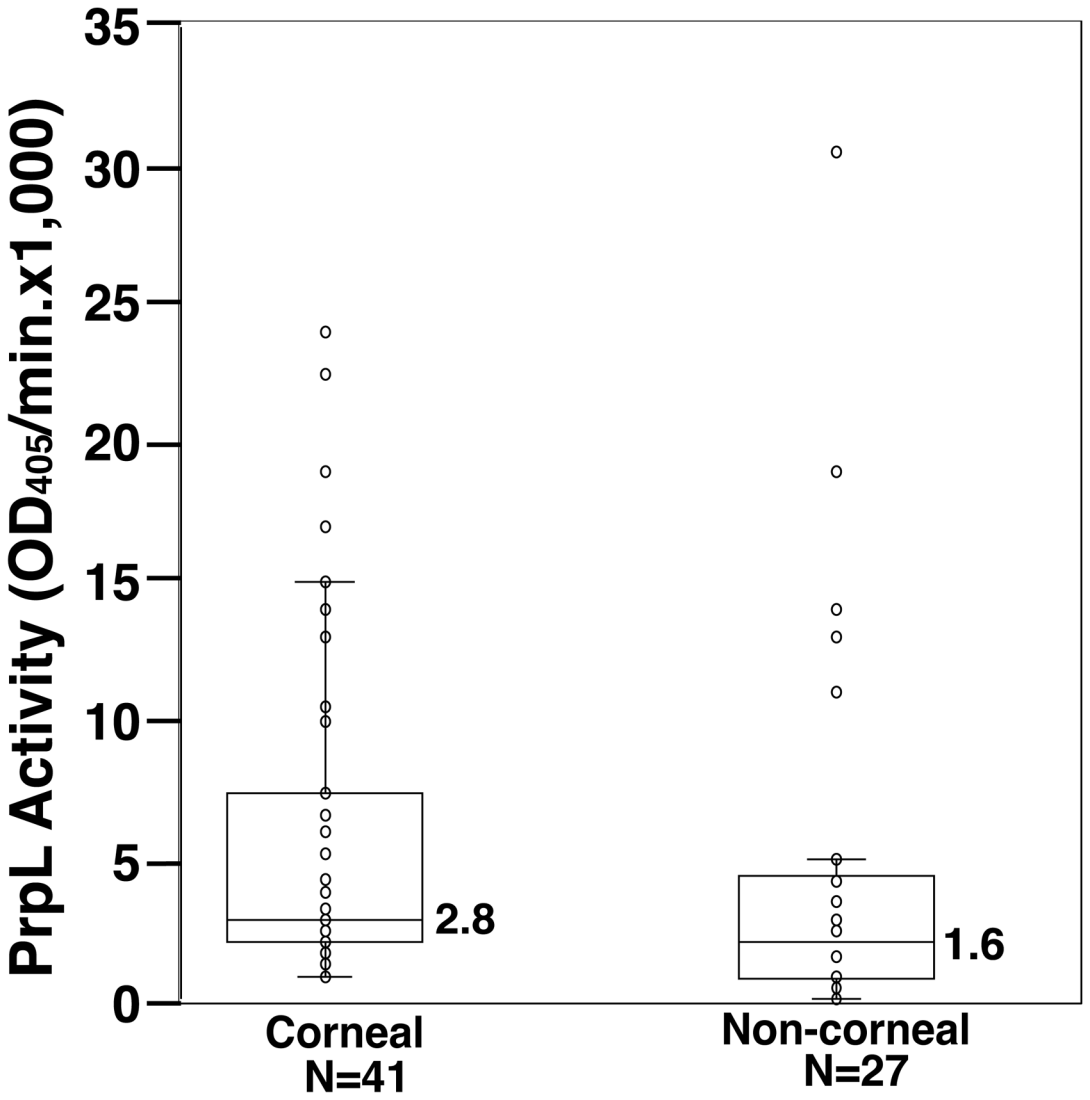
PrpL producing *P. aeruginosa* strains are over-represented in corneal infections. Kinetic analysis of lysyl endopeptidase activity in the culture supernatants from 68 *P. aeruginosa* clinical isolates was determined using S2251 substrate with median OD_405_ value of in corneal (2.8×1,000) versus in non-corneal clinical (1.6×1,000) isolates. OD_405_ determined cleavage of substrate to produce p-nitroaniline that shows maximum absorbance at 405 nm. Mann–Whitney two-tailed U test for asymmetrically distributed data indicated a statistically significant (*p* = 0.035) higher PrpL activity in corneal isolates.

We quantitated PrpL lysyl endopeptidase activity in 41 corneal and 27 non-corneal *Pseudomonas* clinical isolates using the chromogenic substrate S2251. Although activity as high as 320 IU/mL was detected in the supernatants of a few non-corneal isolates, half of the isolates expressed negligible activity (Figure 1). We used PA01 strain, a wound isolate and first sequenced *P. aeruginosa* strain, as the Type strain in this study and grouped the strains based upon their PrpL activities and pyocyanin levels relative to the PA01 strain in Table 1. Interestingly, higher pyocyanin levels than that of PA01 (OD_520nm_ = 0.9) were also detected in the majority of corneal than non-corneal isolates (Table 1). Furthermore, PrpL activity with the median OD_405_ of 2,800 among corneal strains (Figure 1) was significantly greater than that of non-corneal isolates (1,600, *p* = 0.035). Thus, while PrpL activity may not be essential for establishment of corneal infection, association of PrpL production levels with corneal infecting isolates suggests that the protease contributes to destructive keratitis by *P. aeruginosa* in infected individuals.

**Table 1:** Grouping of *P. aeruginosa* strains based upon PrpL activity and pyocyanin levels.

*P. aeruginosa* strains	Number of strains	Total pyocyanin (OD520 ≤ 0.9*)	Total pyocyanin (OD520 > 0.9)
PrpL Vmax per mL (>0.5 to<100)			
(a) Corneal	15	4	11
(b) Non-corneal	16	11	5
PrpL Vmax per mL (100 to 500)			
(a) Corneal	10	1	9
(b) Non-corneal	7	3	4
PrpL Vmax per mL (>500)			
(a) Corneal	16	2	14
(b) Non-corneal	4	0	4
Total	68	21	47

^*^Pyocyanin level in PA01.

### PA64481 strain secretes higher levels of active PrpL protease

**Figure 2:**
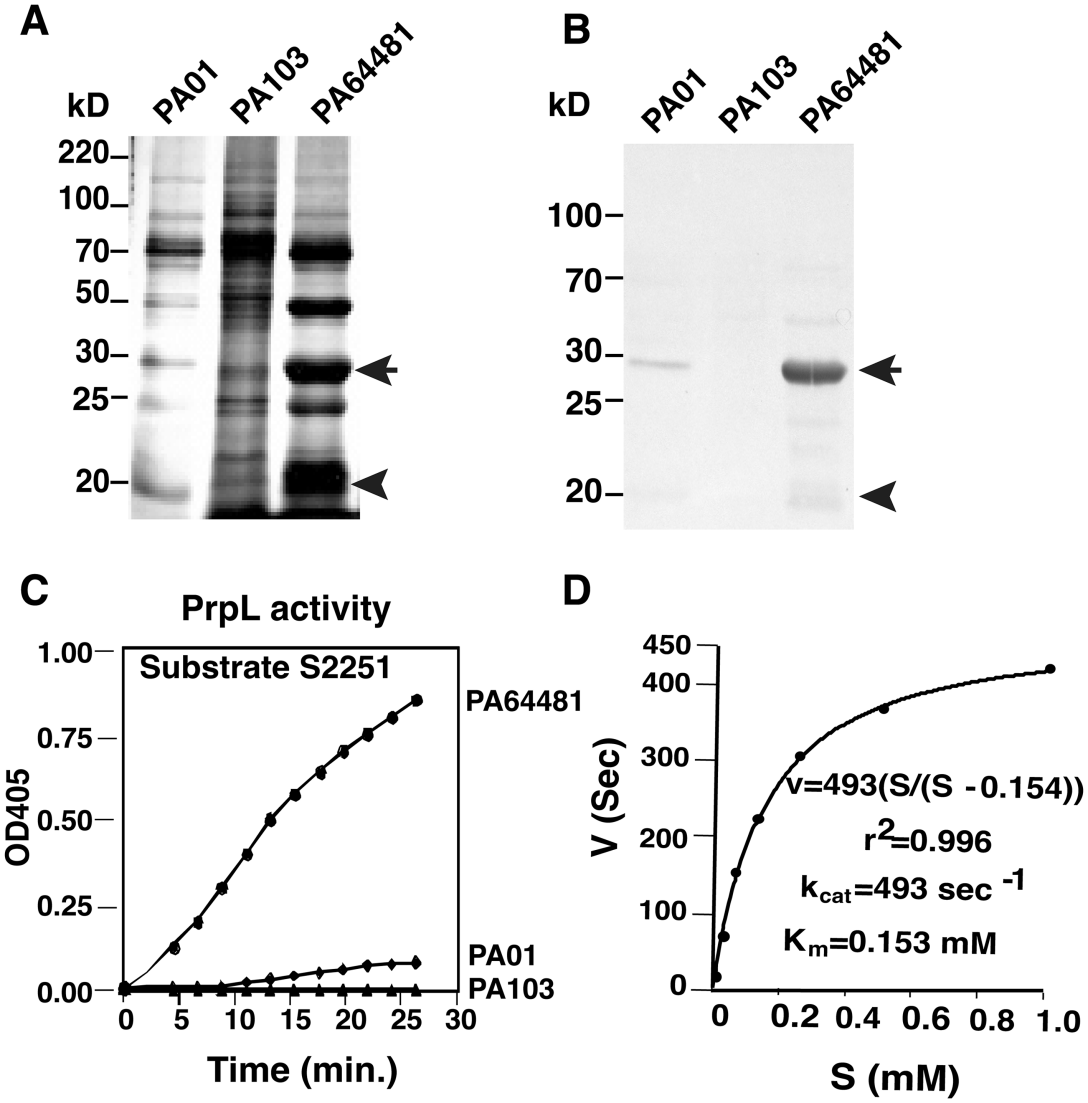
Detection of high specific activity of PrpL in PA64481 strain supernatant. **(A)** Supernatants from ~2 × 10^10^ bacteria were loaded and SDS-PAGE was followed by silver staining (Merril et al., 1981). High secreted PrpL levels in PA64481, moderate level in PA01 were detected while PrpL was undetectable in PA103 strain. **(B)** Immunoblotting using anti-PrpL polyclonal antibodies detected a major ~27 kD PrpL band (arrow) and a minor band due to autocleavage of the PrpL protease (arrowhead). **(C)** Kinetic analysis of lysyl endopeptidase activity using S2251 substrate confirmed that secreted PrpL activity in PA64481 is significantly higher than PA01. No activity was detected in 30 min in PA103 culture supernatant. **(D)** *K*_cat_ and *K*_m_ of PrpL in PA64481 supernatant purified by ion-exchange and size exclusion chromatography were determined. *K*_m_ of secreted PrpL was calculated to be 0.153 mM.

We included the PA01 as well as PA103 in all experiments because PA103 demonstrate almost undetectable PrpL activity. PA103 strain was isolated from patient’s sputum and was originally considered to lack the secreted proteolytic activity; however, its one derivative PA103-29 exhibited intermediate PrpL activity (O'Callaghan et al., 1996). Silver-stained SDS-PAGE gel of culture supernatants revealed a species with an apparent molecular mass of ~27 kD, i.e., the predicted molecular mass of PrpL, that was much more prominent in PA64481 than in PA01 and PA103 (Figure 2A). This finding was reinforced by immunoblotting the three culture supernatants for PrpL (Figure 2B). Interestingly, pronounced PrpL activity in PA64481 resulted in autocleavage of the protease to some extent (Figures 2A,B). Overall, fewer proteins were observed in the culture supernatant of PA64481 (Figure 2A) likely due to the cleavage of many secreted proteins by highly active PrpL protease.

Bacterial supernatants of the wild-type PA103 displayed no PrpL protease activity after 30 min of incubation with the substrate while PA01 showed relatively low activity (Figure 2C), suggesting that these strains secrete only low levels of this protease. Therefore, PA64481 culture supernatant was subjected to chromatographic techniques described previously for the purification of PrpL from strain PA103 (Engel et al., 1998a), and majority of activity was detected only in the ~27 kD species. Sequence determination of the five N-terminal residues (AGYRD) of this purified protein was identical to the N-terminus of previously reported mature PrpL protease (Wilderman et al., 2001; Supplementary material). Using BCA kit, we estimated the concentration of purified PrpL in the SM9 PA64481 supernatant to be 150 ng of protease/ml, while estimated 0.4 mg of total protein was recovered previously after purification from 46 liters of PA103-29 culture supernatant (Engel et al., 1998a). The k_cat_ and K_M_ of PrpL secreted from PA64481, determined using the substrate S2251, were 493 μM sec^−1^ and 153 μM, respectively (Figure 2D). In contrast, the V_max_ and K_m_ of this protease from PA103-29 (an *exoA* derivative of PA103 that secretes slightly higher levels of PrpL than the parental PA103 strain) were 0.74 μM/min and 727 μM, respectively (Engel et al., 1998a). Thus, more PrpL is produced and secreted by PA64481 than by PA103-29 or PA01. Higher k_cat_ of PA64481 PrpL was observed.

Comparison of deduced amino acid PrpL sequences demonstrated that whole protein sequences as well as the active site residues, serine, aspartate and histidine are conserved in all these serine proteases (Supplementary Figure S1A). Thus, activity in the culture supernatant is mainly reflective of PrpL secreted by each strain. Comparison with lysyl- and arginal endopeptidases showed that PrpL showed slightly higher homology to the arginal endopeptidase LeR than to the lysyl endopeptidases AlK and LeK (Supplementary Figure S1B).

### Disruption of cultured corneal epithelial cells is associated with the level of secreted PrpL

**Figure 3:**
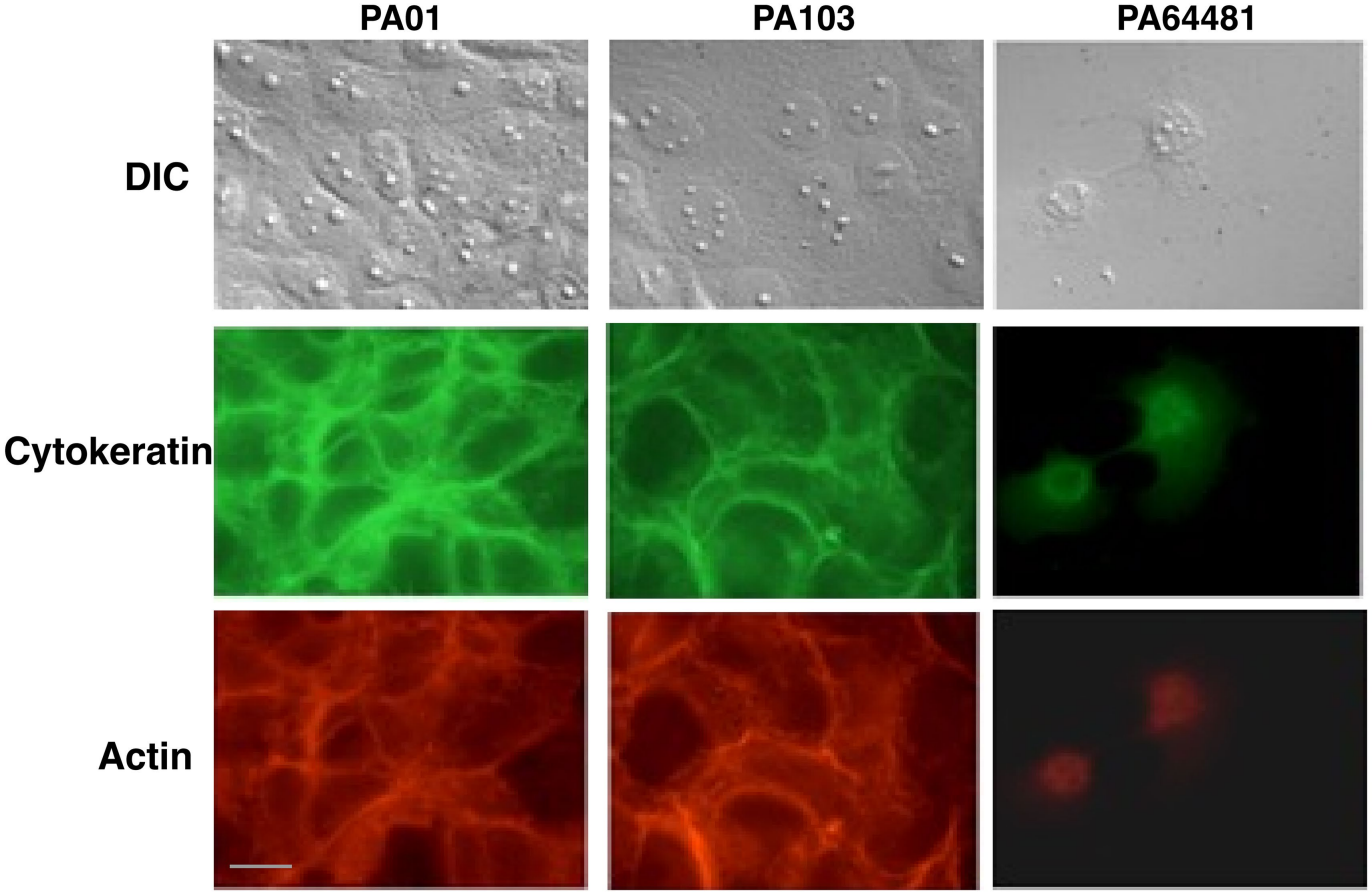
Destruction of HCE-T monolayer and cytoskeleton is associated with secreted PrpL levels in *P. aeruginosa* strains. Corneal cell monolayers on coverslips were treated with culture supernatants at 1:1 dilution in cell culture medium and were fixed at 15 min interval after inactivating protease by PMSF + Aprotinin. The slides were examined by Nomarski (top panels), after staining for cytokeratins followed by detection using FITC-labeled secondary antibody (middle panels), and after staining filamentous actin with TRITC-labeled phalloidin (bottom panels). Disruption of monolayers and the loss of cytoskeleton integrity can be seen within 15 min of treatment with supernatant from PA64481 strain, while the integrity of the cell monolayers was maintained even after 1 h of treatment with both PA01 and PA103 strains supernatants. Bar indicates 10 μm. Fluorescence intensity was quantified from seven independent fields of view after treatment with culture supernatant of each strain using ImageJ. The mean fluorescence intensity± SEM was 62.9 ± 2.7 for PAO1, 46.8 ± 2.5 for PA103, and 5.4 ± 0.5 for PA64481, demonstrating a significant reduction due to erosion of cell monolayer by treatment with PA64481 supernatant.

To understand overall damage caused by PrpL during infection where serpins could be inactivated by pyocyanin, we also determined the levels of this pigment in all strains. We detected higher pyocyanin levels in PA64481supernatant (OD_520_ = 1.7) while levels of this molecule were low to undetectable in PA01 and PA103 strains (OD_520_ 0.9 and 0.2, respectively). We added culture supernatants from three strains to the cultured HCE-T corneal epithelial monolayers. To eliminate the variation due to iron limiting conditions, LB grown bacterial culture supernatants were used to examine the effect of constitutively expressed and secreted PrpL protease. PA64481 supernatant destroyed HCE-T monolayers in less than 15 min (Figure 3, right panel). The cytokeratin organization and filamentous actin network were both disrupted by this treatment. Integrity of HCE-T cell monolayers was unaffected even after 1 h treatment with PA01 and PA103 filter sterilized supernatants (Figure 3, left and central panels) supporting the role of PrpL in corneal damage reported previously. Poor cytokeratin/phalloidin staining suggests that destruction of gap junctions mediated by PrpL could also damage cellular cytoskeleton. We also mention the level of reduction in fluorescence intensity due to destruction of HCE-T monolayers by supernatant of PA64481 in this figure legend and raw data in the Supplementary Figure S2. The role of high pyocyanin levels in PA64481 cannot be defined in a tissue culture system *in vitro* due to the absence of serpins in this system; however, role of PrpL is emphasized because we stopped the further damage by serine protease inhibitors aprotinin and PMSF.

### Inactivation of type II secretion system significantly reduces secretion of PrpL by *Pseudomonas aeruginosa*

**Figure 4:**
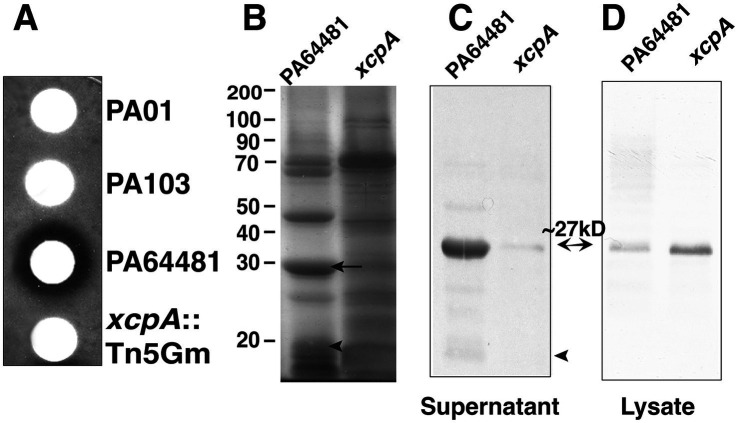
Comparative analysis of PrpL levels and activity in the *xcpA* mutant. **(A)** Caseinase assay using skim milk containing plate overlay with the culture supernatants impregnated filter discs showed no zone of clearance around the mutant in which the *xcpA* gene was disrupted by the Tn5Gm transposon and is comparable to the PA103 strain supernatant, confirming that the *xcpA* disrupted mutant fails to secrete PrpL to a significant level. **(B)** Supernatants from ~5×10^9^ bacteria were loaded and SDS-PAGE was followed by silver staining. The absence of secreted protease in the culture supernatant of the *xcpA* mutant resulted in the accumulation of other secreted proteins as detected by silver staining. **(C)** Immunoblotting using anti-PrpL polyclonal antibodies shows significant reduction in secreted PrpL in the *xcpA* mutant while **(D)** most of the PrpL protease remains associated with bacteria (lysate).

The halo generated by PrpL on casein plates allowed us to determine the level of secreted proteases as indicated by a “halo” around a filter disc impregnated with *P. aeruginosa* culture supernatants. We identified a Tn5Gm insertion mutant of PA64481 that did not produce a halo (Figure 4A). This mutant also did not secrete detectable level of PrpL either by silver staining or immunoblotting (Figures 4B,C). The observed defect was specifically attributed to the lack of secretion because high levels of PrpL was detected in bacterial lysates by Western blotting using anti-PrpL polyclonal antibodies (Figure 4D). Interestingly, the majority of PrpL protease detected by immunoblotting in bacterial lysates was found to be in mature form, i.e., migrated with an apparent molecular mass of ~27 kD (Figure 4D). Sequencing of the DNA fragment flanking the Tn5 revealed transposon insertion into the *xcpA* gene, which encodes an essential component of the type II secretion system (Bally et al., 1991; Bally et al., 1992; Cheng et al., 2023; Kagami et al., 1998). We conclude that PrpL is primarily secreted via the XcpA based type II secretion system of *P. aeruginosa*. During lysis of bacteria, cleavage by other proteases present in the extract (likely periplasmic proteases) could ultimately cleave PrpL pre-protease to produce mature enzyme. Involvement of PrpL in digestion of other secreted proteins of *P. aeruginosa* was further confirmed since protein profile of culture supernatant of *xcpA*: Tn5Gm mutant, which was comparable to that observed in PA103 and not like the parent strain PA64481, a high PrpL secreting strain (Figures 2A, 4B).

**Figure 5:**
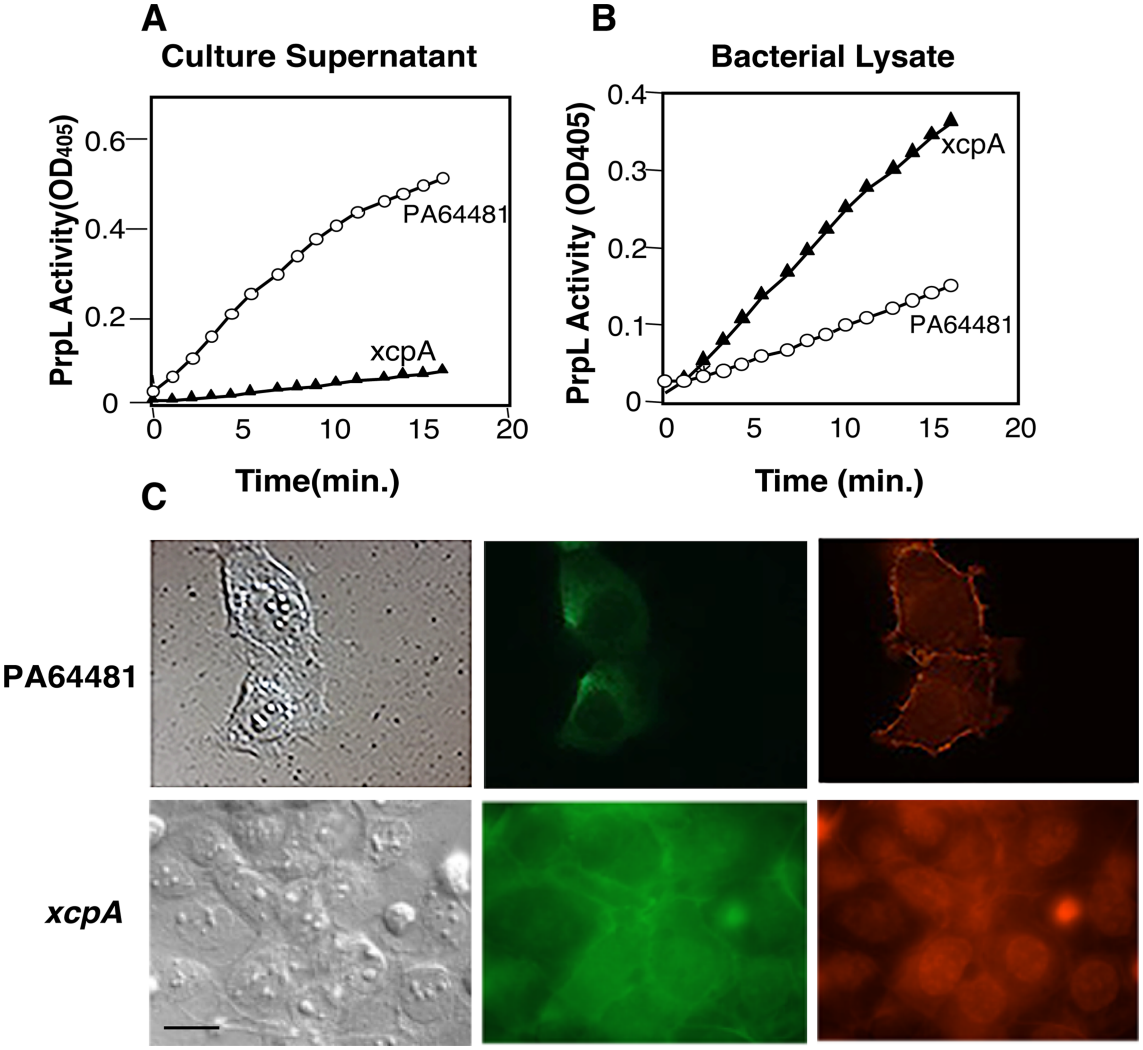
Disruption of human corneal epithelial (HCE-T) cells by supernatant from high PrpL-producing strain PA64481 is eliminated in the *xcpA* mutant. **(A)** Minimal PrpL activity was observed in the *xcpA* mutant culture supernatant compared to that in the wild-type PA64481 strain with S2251 substrate while **(B)** significantly higher lysyl endopeptidase activity in the bacterial lysate than the parental PA64481 strain indicate failure of the mutant to secrete PrpL. **(C)** Corneal cell monolayers on coverglass were treated with culture supernatants at a 1:1 dilution in cell culture medium and were fixed at 15-min intervals after inactivating this serine protease with PMSF+Aprotinin. The slides were examined by Nomarski (left panels), for cytokeratins using FITC-labeled secondary antibody (middle panels). Staining with TRITC-labeled phalloidin labeled filamentous actin (right panels). Disruption of monolayers and the loss of cytoskeleton can be seen within 15 min of treatment with supernatant from the PA64481 strain, while the integrity of the cell monolayers was maintained even after 1 h treatment with the *xcpA* mutant supernatant. Bar indicates 10 μm. Fluorescence intensity was quantified from seven independent fields per treatment using ImageJ. The mean fluorescence intensity ± SEM was 32.9 ± 2.6 for the *xcpA* secretion defective mutant and 5.4 ± 0.5 for PA64481, indicating a significant damage to cell monolayers by PA64481 supernatant.

The results demonstrating PrpL production and secretion were further confirmed by comparing protease activity in PA64481 strain and the *xcpA* mutant culture supernatants (Figure 5A) and lysates (Figure 5B). Interestingly, supernatant from the *xcpA*: Tn5 mutant, which contains only low levels of PrpL, had only a subtle effect on the integrity of monolayers (Figure 5C) and was comparable to that observed after treatment with culture supernatants from PA01 and PA103 strains (Figure 3) as indicated by fluorescence intensity in the figure legend and individual image data from seven fields in Supplementary Figure S2. Pyocyanin level in this mutant (OD_520_ = 1.9) was not affected.

### Characterization of prpL knockout mutant of PA64481 strain

**Figure 6:**
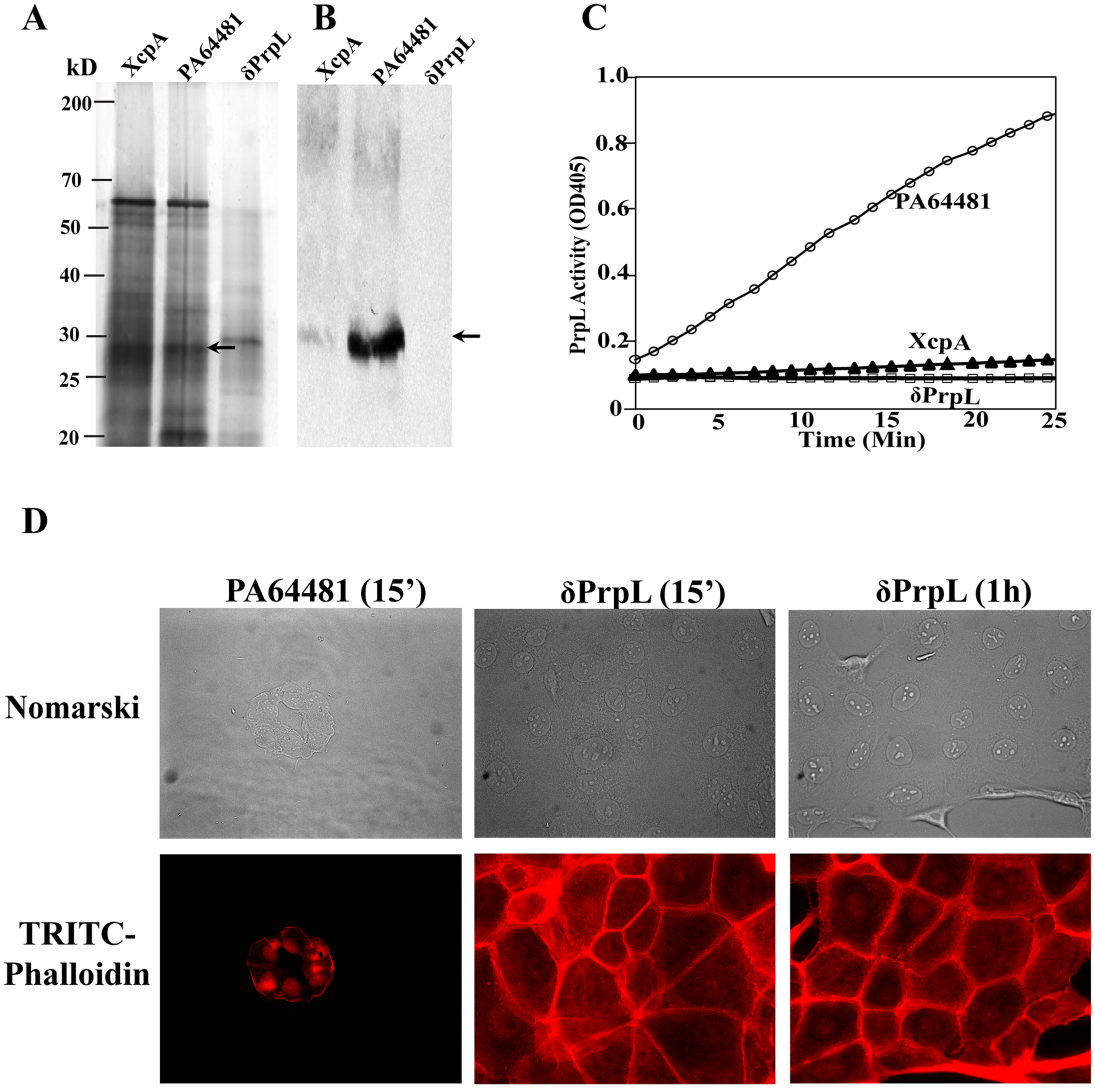
Evaluation of PrpL levels and activity in secreted milieu of the *prpL* mutant and its effect on HCE-T cell monolayer. **(A)** Supernatant from ~2×10^9^ bacteria was loaded and SDS-PAGE was followed by silver staining. The absence of secreted PrpL protease in culture supernatant of the *prpL* mutant analyzed by silver staining was confirmed by **(B)** Western blot analysis (loaded supernatant from equivalent of 1×10^10^ bacteria) using anti-PrpL polyclonal mouse antibodies. **(C)** No PrpL activity was detected in the culture supernatant of the *prpL* mutant using the S2251 substrate. **(D)** After treatment of HCE-T corneal epithelial monolayer with *P. aeruginosa* culture supernatants, slides were examined by Nomarski (top panels), and after staining with TRITC-labeled phalloidin for filamentous actin (bottom panels). Unlike the wild-type PA64481 strain supernatant, secreted protein milieu of the *prpL* mutant failed to destroy the HCE-T cell monolayer even after 1 h of incubation. Fluorescence intensity was measured from seven independent fields per treatment and timepoint using ImageJ. The mean fluorescence intensity± SEM was 30.3 ± 2.1 for δPrpL at 15 min, 30.7 ± 2.1 for δPrpL at 1 h, and 2.3 ± 0.5 for PA64481, indicating a significant damage top corneal cell monolayer caused by PA64481 supernatant.

We cannot rule out the possibility that other secreted proteases that use Type II secretion machinery could also be involved in corneal damage. To evaluate the role of PrpL directly, we generated and characterized *prpL* knockout mutant (δPrpL) in high PrpL producing PA64481 strain (Figure 6). The absence of PrpL in culture supernatant was observed by SDS-PAGE analysis and by Western blotting. Unlike *xcpA* mutant, supernatant of the *prpL* mutant completely lacked serine protease endopeptidase activity using S2251 substrate (Figure 6C), indicating that PrpL is the exclusive active serine protease in this strain that is associated with the cleavage of this substrate. Furthermore, culture supernatant of this mutant did not disrupt HCE-T monolayers (Figure 6D) even after 1 h of incubation, further supporting our data that PrpL is the major protease involved in corneal tissue damage. Again, the level of fluorescence intensity reflects this phenomenon in the legend of this figure and in Supplementary Figure S2.

## Discussion

O’Callaghan and coworkers first identified PrpL (protease IV) and showed its potential role in corneal damage (Caballero et al., 2001a; Engel et al., 1998a; Engel et al., 1998b; Engel et al., 1997; Traidej et al., 2003) because a mutant lacking PrpL activity caused significantly less pathology than the parental strain in corneal scratch mouse model. Although PrpL production showed no discernible effect in a rabbit scratch model (O'Callaghan et al., 1996), severe damage in rabbit intrasomal infection model was reported as time lapsed observation after infection (Engel et al., 1998b; Engel et al., 1997). An experimental hurdle associated with these studies is the fact that they were either performed with low PrpL producing PA103-29 strain or its derivatives.

We show here that corneal isolates of *P. aeruginosa* uniformly secrete PrpL at levels that are significantly (*p* = 0.035) higher than that secreted by noncorneal isolates albeit there were some outliers in the latter. In our assays, PA103 supernatant demonstrated no lysyl endopeptidase activity, even when the assays were extended to 1 h, suggesting exquisite sensitivity of the substrate to PrpL protease. A previous study with a strain with high PrpL activity did not address its possible role in tissue damage or pathogenesis (Elliott and Cohen, 1986).

We show that PA64481 secretes large amounts of PrpL with potent catalytic activity. An insertion in the *xcpA* gene of PA64481 abolished secretion of PrpL but not its expression. Given that XcpA is a peptidase involved in processing of proteins that are suggested to form the type II secretory machinery in the outer membrane of *P. aeruginosa* (Bally et al., 1991; Bally et al., 1992; Cheng et al., 2023; Kagami et al., 1998), our results suggest that PrpL is secreted primarily via this type II secretion machinery similar to lipase and elastases, which are also secreted by the same system (Bally et al., 1992); however, PrpL and not elastase is usually expressed during *P. aeruginosa* infection (Kernacki et al., 1995) and thus, may not play a major role in corneal damage (Hobden, 2002; Pinna et al., 2008; Preston et al., 1997; Twining et al., 1986) or damage to collagen by cleavage and activation of the host pro-matrix metalloproteinases during infection (Nagano et al., 2001). There are three quorum sensing (QS) systems described in *P. aeruginosa* strains. Previously, QS regulatory mechanisms were shown to be involved in the expression of secreted proteases including of PrpL in PA01 strain, which produces limited amount of this protease (Li and Lee, 2019; Oh et al., 2017; Park et al., 2014; Schuster et al., 2003); however, contribution of individual QS system was not clearly defined. In our future studies, we intend to use the specific mutants with disrupted LasR, RhlR and PqsR encoding genes in the high proteases producing and sequenced PA14 strain to determine whether one or more of QS systems regulate PrpL expression and of other proteases.

Corneal damage is a hallmark of serious infections by *P. aeruginosa*, and the activity of PrpL likely contributes to epithelium destruction significantly. Consistent with this notion, we show here that the ability to damage monolayers of corneal epithelial cells is associated with the secretion of PrpL at high levels and the absence of PrpL fails to disrupt HCE-T corneal epithelial cell monolayer observed by the culture supernatant of the wild-type PA64481 strain (Figures 5, 6). Our kinetic analysis using S2251 substrate of the *prpL* mutant supernatant established that this is the only secreted serine protease in this strain of *P. aeruginosa* and is responsible for corneal cell monolayers *in vitro*. Other proteases do not appear to play any discernible role in tissue damage in this experiment.

A role for PrpL may not be restricted to corneal infections because we show here that several non-corneal *P. aeruginosa* isolates expressed detectable levels of lysyl endopeptidase activity. In a previously conducted competition experiments with wild type PA01 and a PA01-derived disruption mutant implicated PrpL in the efficient colonization of rat lungs (Wilderman et al., 2001). It is easy to imagine that higher PrpL protease activity, as well as other secreted *P. aeruginosa* proteases, contributes to the pulmonary injury suffered by patients with acute pneumonia or cystic fibrosis-associated lung disease. Identification of *P. aeruginosa* clinical isolates that produce high levels of PrpL and pyocyanin will facilitate future studies involving evaluation of the role of these molecules in colonization and damage of various tissues during infection. Furthermore, the characterization of various regulatory mechanisms that contribute to the production of both PrpL could help identify overall pathogenesis mechanisms of *P. aeruginosa* and damage in corneal and wound infections in mammalian hosts as incurred by this protease. Although the focus of this study is to determine association of serine protease PrpL with corneal infections, we will determine the levels of production and activities of other secreted proteases in different clinical strains listed in this study and determine their roles in tissue damage by generating specific mutants in high producing strain(s).

## Conclusion

We show here that although PrpL is produced and secreted at significantly high levels in several *P. aeruginosa* isolates from cornea of infected patients and destroyed HCE-T corneal cell monolayers rapidly *in vitro*. We found that some strains isolated from other clinical sites also show high levels of this protease. Interestingly, several of these strains also secreted high levels of pyocyanin pigment, a serpins inhibitor. A mutant disrupting *xcpA* gene almost eliminated PrpL secretion, indicating that Type II secretion system is involved in secretion of this serine protease. The lack of disruption of HCE-T monolayers by both *xcpA* and *prpL* mutants suggests that PrpL, and not any other secreted proteases, are involved in swift destruction of corneal damage. Contribution of PrpL together with other proteases will be explored in other infection models, such as wound and respiratory tract and in *P. aeruginosa* dissemination from biofilms.

## Data Availability

The original contributions presented in the study are included in the article/Supplementary material, further inquiries can be directed to the corresponding author.
