# Supplementary material for: Retrospective analysis of secreted PrpL protease activity in clinical isolates of Pseudomonas aeruginosa and its association with corneal tissue damage
Source: Front Microbiol. 2026 May 8;17:1824817. doi: 10.3389/fmicb.2026.1824817 (PMC13195018; doi:10.3389/fmicb.2026.1824817)
Supplement: Supplementary file 1 [file Supplementary_File_1.pdf]

SUPPLEMEMTARY INFORMATION.

Methods.

Primers based on the known sequence of *prpL* of *P. aeruginosa* strain PA01 [38], 5’ ATGCATAAGAGAACGTACCTGAATG 3’ and 5’TCAGGGCGCGAAGTAGCGGGA 3’ and Hercules polymerase (Stratagene Co. Inc.) were used to amplify, clone and sequence the *prpL* genes from *P. aeruginosa* PA64481, PA01 and PA103. The PCR products were cloned in Topo-XL vector (Invitrogen Inc.) and sequenced. Among 68 strains evaluated in this study, six strains genomes sequencing have been completed with Accession numbers for three major strains: PA01 (QZFY00000000), PA103 (NZ_JAMDFM000000000), PA14 (AY273869).

Supplementary Figure 1A. PrpL protein sequence alignment of three *P. aeruginosa* strains depicting signal peptide (blue), N-terminal sequencing of the mature protein (green), and active site triad residues: His, Asp, Ser (magenta) are marked. Percent identity matrix is shown below.

```
PA01         MHKRTYLNACLVLALAAGASQALAAPGASEMAGDVAVLQASPASTGHARFANPNAAISAA    60
PA103        MHKRTYLNACLVLALAAGASQASAAPGASEMAGDVAVLQASPASTGHARFANPNAATSAA    60
PA64481      MHKRTYLNACLVLALAAGASQALAAPGASEMAGDVAVLQASPASTGHARFANPNAAISAA    60
             ********************** ********************************** ***

PA01         GIHFAAPPARRVARAAPLAPKPGTPLQVGVGLKTATPEIDLTTLEWIDTPDGRHTARFPI    120
PA103        GIHFAAPPARRVARAAPLAPKPGTPLQVGVGLKTATPEIDLATLEWIDTPDGRHTARFPI    120
PA64481      GIHFAAPPARRVARAAPLAPKPGTPLQVGVGLKTATPEIDLTTLEWIDTPDGRHTARFPI    120
             *****************************************:******************

PA01         SAAGAASLRAAIRLETHSGSLPDDVLLHFAGAGKEIFEASGKDLSVNRPYWSPVIEGDTL    180
PA103        SAAGAASLRAAIRLETRSGSLPDDVLLHFAGAGKEIFEASGKDLSLNRPYWSPVIEGDTL    180
PA64481      SAAGAASLRAAIRLETRSGSLPDDVLLHFAGAGKEIFEASGKDLSVNRPYWSPVIEGDTL    180
             ****************:***************************:***************

PA01         TVELVLPANLQPGDLRLSVPQVSYFADSLYKAGYRDGFGASGSCEVDAVCATQSGTRAYD    240
PA103        TVELVLPANLQPGDLRLSVPQVSYFADSLYKAGYRDGFGASGSCEVDAVCATQSGTRAYD    240
PA64481      TVELVLPANLQPGDLRLSVPQVSYFADSLYKAGYRDGFGASGSCEVDAVCATQSGTRAYD    240
             ************************************************************

PA01         NATAAVAKMVFTSSADGGSYICTGTLLNNGNSPKRQLFWSAAHCIEDQATAATLQTIWFY    300
PA103        NATAAVAKMVFTSSADGGSYICTGTLLNNGNPPKRQLFWSAAHCIEDQATAATLQTIWFY    300
PA64481      NATAAVAKMVFTSSADGGSYICTGTLLNNGNSPKRQLFWSAAHCIEDQATAATLQTIWFY    300
             ******************************* ****************************

PA01         NTTQCYGDASTINQSVTVLTGGANILHRDAKRDTLLLELKRTPPAGVFYQGWSATPIANG    360
```

```
PA103       NTTQCYGDASTINQSVTVLTGGANILHRDAKRDTLLLELKRTPPAGVFYQGWSATPIANG   360
PA64481     NTTQCYGDASTINQSVTVLTGGANILHRDAKRDTLLLELKRTPPAGVFYQGWSATPIANG   360
            ************************************************************

PA01        SLGHDIHHPRGDAKKYSQGNVSAVGVTYDGHTALTRVDWPSAVVEGGSSGSGLLTVAGDG   420
PA103       SLGHDIHHPRGDAKKYSQGNVSAVGVTYDGHTALTRVDWPSAVVEGGSSGSGLLTVAGDG   420
PA64481     SLGHDIHHPRGDAKKYSQGNVSAVGVTYDGHTALTRVDWPSAVVEGGSSGSGLLTVAGDG   420
            ************************************************************

PA01        SYQLRGGLYGGPSYCGAPTSQRNDYFSDFSGVYSQISRYFAP------------------   462
PA103       SYQLRGGLYGGPSYCGAPTSQRNDYFSDFSGVYSQISRYFAP------------------   462
PA64481     SYQLRGGLYGGPSYCGAPTSQRNDYFSDFSGVYSQISRYFAP------------------   462
            ******************************************
```

Percent Identity Matrix

PA01	100.00%	98.70%	99.78%
PA103	98.70%	100.00%	98.92%
PA64481	98.92%	99.78%	100.00%

Supplementary Figure 1B. PrpL of *P. aeruginosa* is slightly more closely related by amino acid sequence to *Lysobacter enzymogenes* arginal endopeptidase, LeR than lysyl endopeptidase of the same species.

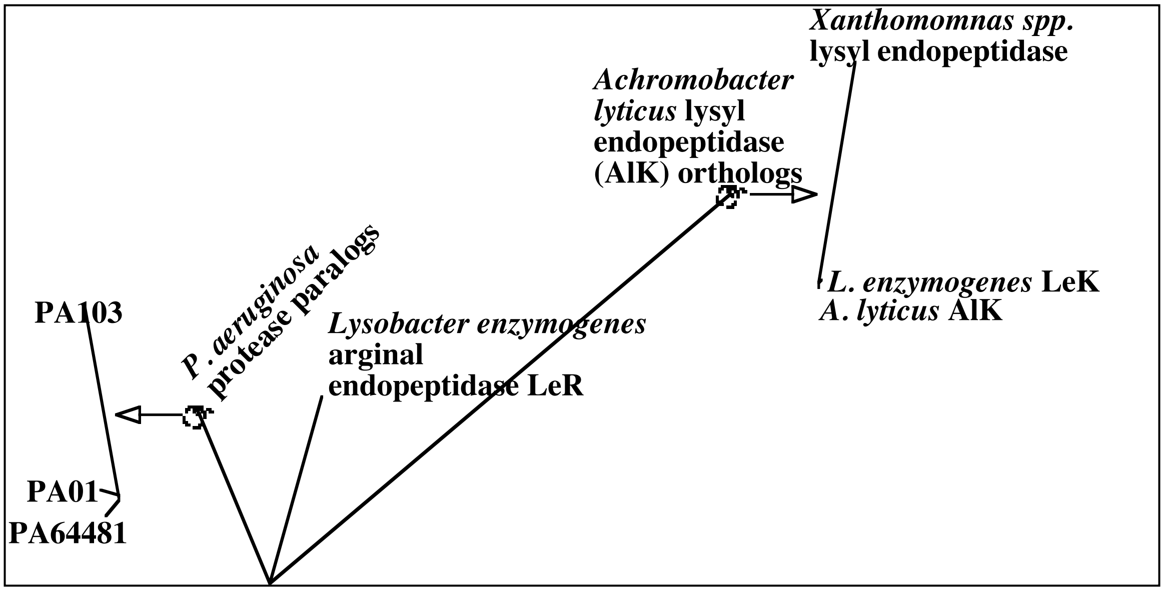

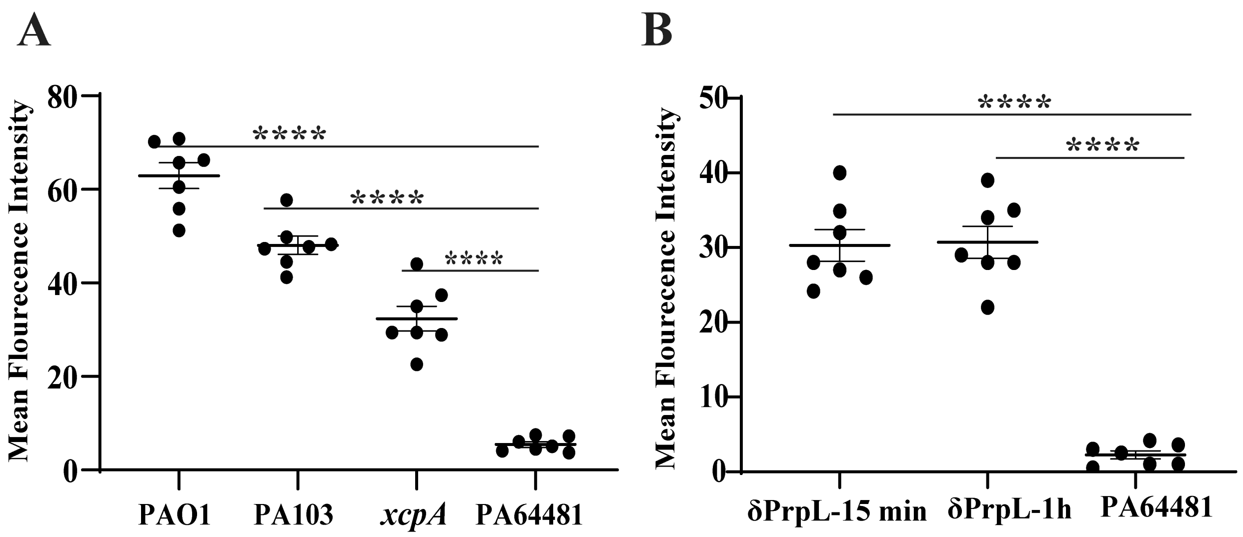

Supplementary Figure S2. Analysis of fluorescence intensity in each image using ImageJ. Fluorescence intensity was measured using ImageJ software from microscopic images acquired under identical exposure settings. Figures S2A and S2B represent data from different experiments. Selected microscopic images for treatment with culture supernatant of each strain are shown in Figures 3, 5 and 6. Data represents mean fluorescence intensity values obtained from seven different fields. Error bars indicate standard error of mean. Statistical analysis was performed using one-way ANOVA, which revealed that PA64481 exhibited a significantly lower mean fluorescence intensity compared to the other strains (**** $p < 0.0001$), mainly because of destruction of the cell monolayer.

Table S1: List of strains and sources

Sr. No.	Strains #	Source
1	PA64481(*xcpA*::Tn5Gm)	
2	CF1	Cystic fibrosis
3	CF2	Cystic fibrosis
4	CF3	Cystic fibrosis
5	CF4	Cystic fibrosis
6	CF5	Cystic fibrosis
7	CF6	Cystic fibrosis
8	CF26	Cystic fibrosis
9	CF27	Cystic fibrosis

10	CF29	Cystic fibrosis
11	CF30	Cystic fibrosis
12	PA14	Cystic fibrosis
13	PA27853	ATCC
14	PA3227	Urine
15	PA40956	Sputum
16	PA32104	Blood
17	PA64481	Sputum
18	PA46273-1	Sputum
19	PA45402	Sterility
20	PA4606	stool
21	PAC180	Coburn-Lory-noncorneal
22	PA103	Coburn-Lory-noncorneal
23	PA388	Coburn-Iglewski-noncorneal
24	PADGI	Coburn-Iglewski-noncorneal
25	PAPAK	Coburn-Lory-noncorneal
26	PA01	Noncorneal
27	PA23493	Urine
28	PA69660 / 19660	Non-Croneal
29	BPEI1	Corneal
30	BPEI2	Corneal
31	BPEI3	Corneal
32	BPEI4	Corneal
33	BPEI5	Corneal
34	BPEI6	Corneal
35	BPEI7	Corneal
36	BPEI8	Corneal
37	BPEI9	Corneal
38	BPEI10	Corneal
39	BPEI11	Corneal
40	BPEI12	Corneal
41	BPEI13	Corneal
42	BPEI14	Corneal
43	BPEI15	Corneal
44	BPEI16	Corneal
45	BPEI17	Corneal
46	BPEI18	Corneal
47	BPEI19	Corneal
48	BPEI20	Corneal
49	BPEI21	Corneal

50	BPEI22	Corneal
51	BPEI23	Corneal
52	BPEI24	Corneal
53	BPEI25	Corneal
54	BPEI26	Corneal
55	BPEI27	Corneal
56	BPEI28	Corneal
57	BPEI29	Corneal
58	BPEI30	Corneal
59	BPEI31	Corneal
60	PA6073	Corneal
61	PA6077	Corneal
62	PA6206	Croneal
63	PA6294	Corneal
64	PA6354	Corneal
65	PA6382	Corneal
66	PA6389	Corneal
67	PA6436	Corneal
68	PA6452	Corneal
69	PA6487	Corneal
